# Thermischer Einfluss urbaner Untergrundstrukturen auf die Grundwassertemperaturen im Kanton Basel-Stadt

**DOI:** 10.1007/s00767-021-00483-1

**Published:** 2021-05-27

**Authors:** Dominic Becker, Jannis Epting

**Affiliations:** Angewandte und Umweltgeologie, Departement Umweltwissenschaften, Universität Basel, Bernoullistr. 32, 4056 Basel, Schweiz

**Keywords:** Grundwassererwärmung, Temperaturmessungen, Tiefgaragen und Tunnelbauwerke, Wärmelasten Untergrundstrukturen, COVID-19-Pandemie, Elevated groundwater temperatures, Temperature measurements, Underground parking and tunnels, Thermal loads of subsurface structures, COVID-19 pandemic

## Abstract

In Basel (CH) wurde mit Monitoringsystemen der thermische Einfluss unterschiedlicher Untergrundstrukturen, einschließlich fünf Tiefgaragen sowie einem Autobahntunnel, auf die urbanen Grundwasserressourcen untersucht. Die Daten wurden anschließend mit gemessenen Meteo- und Grundwassertemperaturdaten sowie Resultaten einer Wämetransportmodellierung zusammenhängend ausgewertet.

In den Tiefgaragen wurden auch über die Wintermonate deutlich erhöhte durchschnittliche Temperaturen zwischen 18,8 und 21,1 °C erfasst. Über den weitaus größten Zeitraum emittieren die Tiefgaragen somit Wärme in den Untergrund. Die Messdaten im Autobahntunnel hingegen deuten darauf hin, dass in den Sommermonaten zwar auch Wärme in den Untergrund emittiert, im Winterhalbjahr aber Wärme aus dem Untergrund absorbiert wird.

Zudem zeigen die Temperaturverläufe in den Tiefgaragen eine klare Abhängigkeit von der Nutzungsart: bei höherem Aufkommen täglicher Ein- und Ausfahrten konnten größere tägliche Temperaturanstiege nachgewiesen werden, mit Unterschieden von bis zu 2 °C in den Tagesmittelwerten. Besonders deutlich wird dies im Zeitraum des „Lockdowns“ während der COVID-19-Pandemie zwischen März und Mai 2020.

## Abstract (en)

In Basel (CH), the thermal impact of various subsurface structures on urban groundwater resources, including five underground parking lots and a freeway tunnel, were investigated by monitoring systems. Data were analyzed together with meteorological and groundwater temperature data and results from heat-transport modelling.

Significantly elevated temperatures between 18.8 and 21.1 °C were recorded in the underground parking lots, even in winter. Thus, underground parking lots emit heat into the surroundings all year. In comparison, data recorded in the freeway tunnel indicate that in the winter months heat can also be absorbed from below ground.

In addition, the temperatures of underground parking lots show a clear dependence on the type of use: with a higher number of daily entrances and exits, greater daily temperature increases were detected, with differences of up to 2 °C. This became particularly clear in the “lockdown” period during the COVID-19 pandemic between March and May 2020.

## Einleitung

Untersuchungen zu erhöhten urbanen Grundwassertemperaturen und der Subsurface Urban Heat Island (SUHI)-Effekt sind mittlerweile ein etablierter Forschungsbereich (z. B. Ferguson und Woodbury 2007; Zhu et al. 2010; Menberg et al. 2013a; Epting and Huggenberger 2013). Es existieren jedoch nur wenige Arbeiten, welche eine differenzierte Betrachtung des thermischen Einflusses von Untergrundstrukturen behandeln. Eine Kombination von verschiedenen Einflussfaktoren wie die zunehmende Bodenversiegelung und die thermische Nutzung des Untergrundes, aber vor allem auch wärmeemittierende Untergrundstrukturen wie Tunnelbauten und Tiefgaragen resultieren in einem nachweislichen Anstieg der basel-städtischen Grundwassertemperaturen von bis zu 9 °C (Epting et al. 2013, 2017a, b; Mueller et al. 2018). Vorausgehende Analysen, die in der Stadt Basel (Schweiz) durchgeführt wurden, haben bereits gezeigt, dass in urbanen Gebieten der anthropogene thermische Einfluss beheizter Untergrundstrukturen größer ist als die zu erwartenden Auswirkungen des Klimawandels (Epting und Huggenberger 2013).

Stadtentwicklung wird zunehmend auch im Untergrund stattfinden (z. B. Bobylev 2009), thermische Beeinträchtigungen urbaner Untergrundressourcen werden vermehrt zu Konflikten zwischen den verschiedenen Nutzern und thermische Kontaminationen werden zwangsläufig zu einer Verringerung der Grundwasserqualität führen (z. B. Possemiers et al. 2014). Doch bisher ist wenig über die biologischen, chemischen und physikalischen Aspekte des Grundwassers und über den Einfluss erhöhter Temperaturen auf die Grundwasserqualität bekannt (CEC 2000; Bates et al. 2008; Brielmann et al. 2009; Jesußek et al. 2013; Kipfer und Livingstone 2008). Die Auswirkungen von SUHI auf die Untergrundtemperaturen entwickeln sich somit zu einem globalen Grundwasserqualitätsproblem.

Der Wärmeverlust von Untergrundstrukturen über das Erdreich kann bis zu 50 % der jährlichen Wärmelast eines Gebäudes betragen (z. B. Deru 2003). Mit zunehmend effizienter werdender oberirdischer Gebäudeisolation gewinnt eine detaillierte Betrachtung unterirdischer Wärmeverluste vermehrt an Bedeutung. Bereits in vorausgegangenen SUHI-Studien wurden Tiefgaragen als Quellen für Anomalien der Grundwassertemperaturen diskutiert (Epting 2017; Iskander et al. 2001; Menberg et al. 2013b; Tissen et al. 2019; Zhu et al. 2010).

Im Allgemeinen berücksichtigen die meisten Untersuchungsansätze nur einzelne unterirdische Strukturen. So verwendete Ampofo et al. (2006) beispielsweise numerische Modelle, um die Wärmelast einer U‑Bahn zu untersuchen, wobei bis zu 30 % der Wärmelast der U‑Bahn in den Untergrund eingetragen werden kann. Dědeček et al. (2012) zeigten, dass für zwei Orte in Mitteleuropa (Prag-Spořilov/Tschechien und Šempeter/Slowenien) das thermische Regime im Untergrund sowohl durch die jüngsten regionalen Klimaveränderungen als auch durch thermische Effekte lokaler anthropogener Strukturen stark beeinflusst wird. Obwohl Dahlem (2000) bereits feststellte, dass der Wärmeverlust durch die advektive Grundwasserströmung im Vergleich zu rein konduktiven Wärmeverlusten einen Faktor 10 ausmachen kann, untersuchten nur wenige Studien den Einfluss der Grundwasserströmung auf den Wärmeverlust beheizter Gebäudestrukturen. Auf städtischer Ebene haben Menberg et al. (2013a) und Benz et al. (2015) analytische Wärmeflussmodelle und einen GIS-Ansatz (Geographisches Informationssystem) verwendet, um zu zeigen, dass Untergrundstrukturen einen signifikanten Anteil der gesamten anthropogenen Wärmelast von urbanen Grundwasserleitern ausmachen. Epting et al. (2017a) präsentierten eine systematische Bewertung der thermischen Auswirkungen von unterirdischen Gebäudestrukturen auf städtische Grundwasserressourcen. Ein wesentliches Fazit dieser Arbeit war, dass thermische Auswirkungen von Untergrundstrukturen gemeinhin unterschätzt werden, v. a. auch wegen eines Mangels an Informationen und zuverlässigen Daten.

Im Rahmen der Untersuchungen wurde der thermische Einfluss von urbanen Untergrundstrukturen im Stadtgebiet von Basel evaluiert. Der Fokus lag dabei auf Tiefgaragen und Tunnelbauwerken, welche in die grundwassergesättigte Zone hineinragen und somit einen direkten thermischen Einfluss auf das Grundwasser haben. Die über 1 Jahr kontinuierlich aufgezeichneten Daten der Temperaturen in den Tiefgaragen T_TG_ und dem Autobahntunnel werden im Zusammenhang mit meteorischen Temperaturdaten (T_meteo_) zweier Wetterstationen, Temperaturdaten (T_GW_) von neun Grundwassermessstellen (GWM) sowie Resultaten einer Wämetransportmodellierung (T_GWsim_) ausgewertet.

## Untersuchungsgebiet

**Figure 1:**
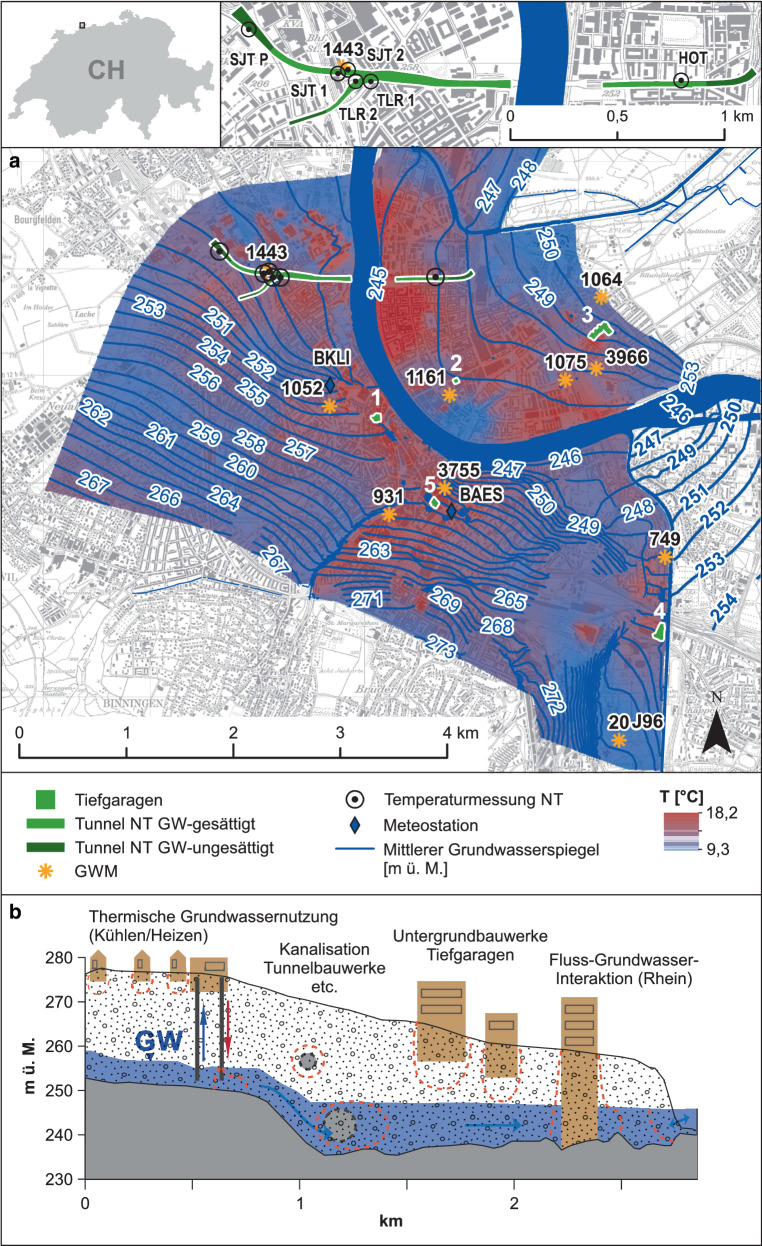

Die untersuchten Untergrundstrukturen liegen im Stadtgebiet von Basel (24 km^2^). Der Kanton Basel-Stadt befindet sich im Nordwesten der Schweiz auf ca. 260 m ü. M., grenzt im Norden an Deutschland und Frankreich und im Süden an die Ausläufer des Falten- und Tafeljuras. Der Rheingraben als alluvialer Ablagerungsraum umfasst die pleistozänen Niederterrassenschotter des Rheins, der Wiese und der Birs, welche auf wasserstauendem Septarienton und Elsässer Molasse liegen (Bitterli-Brunner und Fischer 1988). Die Grundwassermächtigkeiten liegen zwischen 4 und 12 m, der Flurabstand beträgt zwischen 5 und 12 m, und Grundwasserfließgeschwindigkeiten liegen im Bereich von 0,04 und 4 md^−1^ (Epting et al. 2017b). Die regionale Grundwasserfließrichtung folgt dem hydraulischen Gradienten in Richtung der Vorfluter Rhein, Birs, Birsig und Wiese. Es herrschen also größtenteils Grundwasser-exfiltrierende Verhältnisse; während Hochwasserereignissen kann aber auch Oberflächenwasser in den Grundwasserleiter infiltrieren (Abb. 1).

Das T_meteo_-Jahresmittel beträgt, gemäß der Klimanormwerte des Zeitraumes 1981 bis 2010 10,5 °C, allerdings mit steigender Tendenz, sodass mit 12,3 °C im Jahr 2019 das Maximum seit Aufzeichnungsbeginn verzeichnet wurde; die mittlere Jahresniederschlagssumme beträgt rund 850 mm (Basel-Stadt 2020; Klimanormwerte 1981–2010: Lufttemperatur 2 m, MeteoSchweiz; 1981–2010: Niederschlagssumme, MeteoSchweiz). Damit zählt Basel zu einer der wärmsten und niederschlagsärmeren Regionen der Schweiz.

## Methodik

### Gebäudestrukturen im Untergrund von Basel

Auf Grundlage von vorausgehenden Arbeiten und der systematischen Dokumentation von Gebäudestrukturen im Untergrund von Basel (Epting et al. 2017a) konnten insgesamt fünf Tiefgaragen, welche in die grundwassergesättigte Zone reichen, ausgewählt werden (Abb. 1 und Tab. 1). Die in einem GIS organisierten Daten von 3016 Gebäudestrukturen im Untergrund von Basel enthalten sowohl Informationen zu den Geometrien der Untergrundstrukturen, der Gebäudenutzung als auch zur mittleren und maximalen Grundwasserhöhe aus Messungen der letzten 20 Jahre. Ebenfalls wurden die Differenzen zwischen den Grundwasserhöhen und Unterkantentiefen an den jeweiligen Standorten der Untergrundgebäudestrukturen erfasst.

**Table 1:** 

Standort	Tiefe Messystem unter GOK [m]	Gebäudegrundfläche [m^2^]	GWM	Settings und Nutzung
	GOK [m ü. M.]	Kontaktfläche Untergrund [m^2^]	GW-Zustrom	
	Mittlerer GW-Stand [m ü. M.]	Kontaktfläche GW [m^2^]	GW-Abstrom	
	Gebäudetiefe [m ü. M.]			
1: Parkhaus Storchen 2’611’109/1’267’605	−5,1	1’574	Bernoullianum (1052)	Grossbasler Innenstadt nahe Rheinufer 00:00–24:00 Uhr: Wochen‑, Sonn- und Feiertage 142 Kurzzeitstellplätze; während Ladenöffnungszeiten (Mo–Sa) komplett belegt; 800 täglichen Ein- und Ausfahrten
	255,4	44’768	2’610’681/1’267’705	
	ca. 253,3 (GW-Mächtigkeit ca. 5 m)	29’995	Keine Messstelle GW-Abstrom	
	246,8 (anstehender Fels bei 248,2)			
2: Tiefgarage Clarastrasse 2’611’855/1’267’951	−9,6	957	Waldshuterstrasse (1064)	Zentrale Lage Kleinbasler Innenstadt Dauermietende, gewöhnlich wenig Verkehr
	256,1	32’645	2’613’221/1’268’751	
	245,9	4’041	Dolderweg (1161)	
	244,8 (oberhalb anstehender Fels 237,8)		2’611’783/1’267’826	
3: Autoeinstellhalle St. Claraspital 2’613’160/1’268’389	−8,5 und −11,2	422	Waldshuterstrasse (1064)	Tiefendifferenzierte Messung im 2. und im 3. UG
	259,9	34’653	2’613’221/1’268’751	Hirzbrunnenquartier nahe Gemeindegrenze Riehen
	249,4	0 und 4’905	Wettsteinallee 175 (1075)	89 Parkplätze für Angestellte und Besuchende, unter der Woche tagsüber sämtliche Plätze zwei bis dreifach neu belegt, nachts steht es bis auf wenige Dauermietplätze und Fahrzeuge von Nachtschichtangestellten leer
	248,6 (oberhalb anstehender Fels 241,3)		2’612’875/1’267’963	Das unterste Stockwerk wird hauptsächlich vom Personal genutzt, weshalb dort wochen- und feiertags ebenfalls eine signifikant kleinere Anzahl an Plätze belegt ist
			Magdenweglein 46 (3966)	
			2’613’161/1’268’064	
			Zwei Messstellen GW-Abstrom	
4: Tiefgarage St. Jakob-Park 2’613’763/1’265’596	−7,4	8’094	G80-Karussell (20J96)	Östliches Grossbasel, Kantonsgrenze zu Basel-Landschaft, die streckenweise durch die Birs gebildet wird 680 Parkplätze unterhalb des Fussballstadions stehen tagsüber vor allem Kundschaft des Shoppingcenters bzw. Gästen von Fussballspielen zur Verfügung, nachts ist das Parkhaus geschlossen
	259,3	114’420	2’613’376/1’264’591	
	255,1	59’802	Redingstrasse (749)	
	250,3 (anstehender Fels bei 249,7)		2’613’817/1’266’304	
5: Parkhaus Anfos 2’611’652/1’266’823	−16,0	1’702	Heuwaage (931)	Grossbasler Innenstadt und dem Bahnhof SBB und verfügt über 166 Stellplätze. Das unterste Stockwerk steht Dauermietenden zur Verfügung, die oberen tagsüber für Kurzzeitparking
	270,5	57’412	2’611’226/1’266’714	
	257,6 (Flurabstand ca. 3,6 m Tiefe)	12’155	Brunngässlein (3755)	
	252,6 (bis anstehender Fels bei 254,0)		2’611’754/1’266’922	

*GOK* Geländeoberkante

Neben den Messungen von T_TG_ konnten auch Messdaten aus den Autobahntunneln der Nordtangente in die Untersuchungen mit einbezogen werden. Insgesamt wurden hier an sechs Messstandorten Temperaturdaten der Tunnelinnenluft aufgezeichnet. Jeweils zwei Messsensoren an der Stammlinie (Fahrtrichtung Deutschland nach Frankreich und Frankreich nach Deutschland), eine am Tunnelportal des St. Johanns-Tunnels (SJT; Fahrtrichtung Frankreich nach Deutschland), zwei an Auf- und Abfahrt des Tunnelanschlusses Luzernerring (TLR) und eine an der Stammlinie des Horburgtunnels auf der Kleinbasler Rheinseite (Fahrtrichtung Deutschland nach Frankreich). Etwa 2 km der Tunnelstrecke liegen in der grundwassergesättigten Zone (Abb. 1).

### Messinstrumente

Die Erfassung der Temperaturdaten erfolgte mit fünf Datenloggern des Typs EA WLAN-TH+ sowie einem EA WLAN-T+ der Marke Display Visions der Firma Electronic Assembly GmbH aus Gilching, Deutschland. Die Messsysteme erfassen die Temperatur in zehnsekündigen bis zwölfstündigen Messintervallen mit einer Genauigkeit von ±0,2 °C und einer Auflösung von 0,01 °C. Die Verarbeitung der Daten wurde durch das Programm EasyLog WiFi von Lascar electronics vorgenommen, welches die Visualisierung und den Export der Daten als Microsoft Excel-Tabelle oder als Text-Datei (CSV) ermöglichte.

Für die Tiefgaragen wurden kontinuierlich über die Dauer eines Jahres zwischen Dezember 2019 und Dezember 2020 stündliche Mittelwerte der Temperaturen aufgezeichnet. Im Autobahntunnel Nordtangente in Basel wurden an den sechs Messstandorten mit den gleichen Messsystemen und der gleichen Auflösung von Dezember 2018 bis November 2019 Temperaturen aufgezeichnet.

### 3D-Simulation der Grundwasserströmung und -temperatur

Eine weitere Grundlage für die Auswertungen sind 3D-Simulationen der Grundwasserströmung und des Wärmetransports (FEFLOW; Diersch 2014), welche im Rahmen des BFE-Projektes (Schweizer Bundesamt für Energie) „Ist-Zustand und Temperatur-Entwicklung Schweizer Lockergesteinsgrundwasservorkommen“ stattgefunden haben und unter anderem in Mueller et al. (2018) publiziert wurden. Im Rahmen dieses Projektes wurde für das Stadtgebiet von Basel, basierend auf hochaufgelösten tiefendifferenzierten Temperaturmessungen und 3D-Simulationen, der Istzustand des hydraulischen und thermischen Grundwasserregimes für den Zeitraum von 2010 bis 2015 abgeleitet (Abb. 1). Für die hier vorgestellten Auswertungen wurden die Simulationsresultate der T_GWsim_ an den jeweiligen Koordinaten inkl. Tiefe der Messstandorte extrahiert.

### Meteodaten

Grundlage für die meteorischen Temperaturmessungen (T_meteo_) lieferte die Meteodatenbank der Forschungsgruppe „Meteorology Climatology Remote Sensing (MCR)“ der Universität Basel. Für die zusammenhängenden Auswertungen mit den T_TG_ dienten die Daten der beiden Messtationen BKLI und BAES (Abb. 1), welche laut MCR die repräsentativsten Referenztemperaturen für die Stadt Basel liefern, da sie den geringsten Interferenzen wie Strahlungseinflüssen oder fehlender Belüftung ausgesetzt sind. Die Messstation BKLI ist auf dem Dach des Geographie-Gebäudes der Universität Basel an der Klingelbergstrasse 27 installiert und zeichnet (u. a.) Temperaturdaten in 39 m Höhe auf, die Messstation BAES befindet sich auf dem Dach des Turmhauses am Aeschenplatz 2, Daten werden hier in 40 m Höhe erfasst (Feigenwinter et al. 2017). In dieser Arbeit wurden Stundenmittel-Messungen der beiden Standorte für den Messzeitraum der T_TG_-Messungen verwendet, um potenzielle Korrelationen zwischen T_meteo_ und T_TG_ zu untersuchen.

### Grundwassertemperaturdaten

Die Grundwasserstände und -temperaturen werden vom Amt für Umwelt und Energie, Kanton Basel-Stadt (AUE BS), durch ein Beobachtungsnetz von 85 GWM (davon fünf der Industriellen Werke Basel (IWB) und eine des Bundesamts für Umwelt (BAFU)) kontinuierlich überwacht und aufgezeichnet (AUE 2019). In dieser Arbeit wurden die Daten von acht GWM des AUE BS sowie ein Datensatz vom Amt für Umweltschutz und Energie, Kanton Basel-Landschaft (AUE BL), verwendet (Tab. 1). Die Auswahl dieser neun GWM richtete sich primär nach der Orientierung der jeweiligen Standorte zur Position der Tiefgaragen im hydraulischen Gradienten (Abb. 1); bestenfalls konnte für jeden Tiefgaragenstandort mindestens eine GWM aufwärts des Grundwasserstromes sowie eine stromabwärts in die Datenauswertung einbezogen werden. Bedingt durch die Lage der ausgewählten Tiefgaragen war dies nicht für alle Standorte möglich; zwischen der Tiefgarage Storchen und dem Vorfluter (Rhein), in welchen das Grundwasser exfiltriert, existieren keine GWM. Für die Interpretation der Temperaturmessungen der Tunnelinnenluft des Autobahntunnels Nordtangente dienten die Daten der GWM Saint-Louis-Strasse 2 (1443, Abb. 1).

#### Statistische Datenverarbeitung

Um die erfassten Messdaten in einen überschaubaren und interpretationsfähigen Rahmen zu bringen, wurden mit Microsoft Excel aus den CSV-Dateien bereinigte Datensätze als Ausgangsprodukt für weitere Bearbeitungen erstellt. Die statistische Verarbeitung und Visualisierung der Daten erfolgte in der integrierten Entwicklungsumgebung RStudio mit der statistischen Programmiersprache *R*. Lineare Zusammenhänge von Temperaturschwankungen und lokalen Extrema der T_meteo_ und T_TG_ konnten durch Analyse der Korrelationskoeffizienten r nach Pearson berechnet werden, welcher definiert ist durch:

1 $$r_{x,y}=\frac{\sum _{i=1}^{n}\left(x_{i}-\overline{x}\right)-\left(y_{i}-\overline{y}\right)}{\sqrt{\sum _{i=1}^{n}\left(x_{i}-\overline{x}\right)^{2}}\mathrm{*}\sqrt{\sum _{i=1}^{n}\left(y_{i}-\overline{y}\right)^{2}}}$$

mit $n$ dem Stichprobenumfang, $x_{i}$ und$y_{i}$ den Werten und den Mittelwerten $\overline{x}$ und $\overline{y}$ der Variablen $x$ und $y$. Der Pearson-Korrelationskoeffizient r nimmt einen Wert zwischen −1 und 1 an. Ein Wert kleiner 0 deutet auf eine negative Korrelation sowie einen linearen Zusammenhang höherer x‑Werte mit niedrigeren y‑Werten hin, Werte größer 0 deuten auf eine positive Korrelation sowie einen linearen Zusammenhang zwischen höheren Werten von x mit höheren Werten von y hin. Werte von r$\approx$ 0 deuten auf einen fehlenden linearen Zusammenhang der x‑ und y‑Werte hin. Gerade bei Temperaturreihen kommt es vor, dass der Korrelationskoeffizient durch Trends und Saisonabhängigkeit verfälscht wird und vermeintlich höher ausfällt, als er eigentlich ist. Aus diesem Grund wurden die Korrelationskoeffizienten unter Einfluss verschiedener Faktoren getestet: Die unveränderten Datensätze, mit zeitlichem Versatz um Stunden, um eine allfällige verzögerte Reaktion der Temperaturschwankungen in den Tiefgaragen festzustellen, sowie das gleiche Verfahren mit Tagesmittelwerten und zeitlicher Verschiebung um Tage; die Datensätze, ihrer linearen Trends bereinigt, um die Beziehungen zeitlich kleinräumiger Temperaturschwankungen zwischen T_TG_ und T_meteo_ zu untersuchen, ebenfalls mit den gemessenen Stunden- und berechneten Tagesmitteltemperaturen. Zur „Enttrendung“ der Temperaturzeitreihen wurde für jeden Zeitpunkt ein Wert des linearen Gradienten extrahiert und von den Messtemperaturen desselben Zeitpunktes abgezogen.

## Resultate

### Temperaturmessung Tiefgaragen

**Figure 2:**
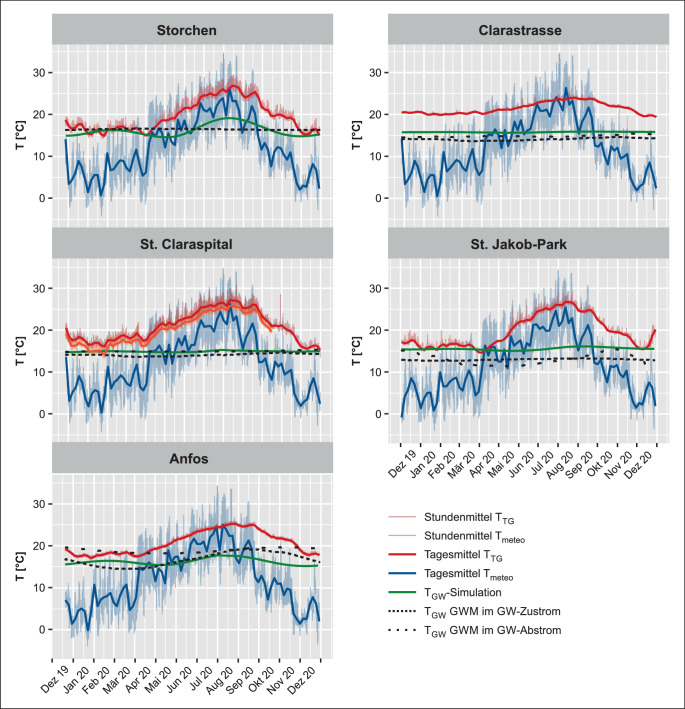

Zur Verdeutlichung von Trends und Zusammenhängen in größerem Rahmen sind sowohl die tatsächlich gemessenen stündlichen Temperaturwerte als auch die gleitenden Mittelwerte von T_TG_ und T_meteo_ dargestellt. Die Temperaturdaten der kantonalen GWM wurden, wenn möglich, in Abhängigkeit ihrer Position im Grundwasserzu- oder -abstrom zur jeweiligen T_TG_-Messstelle abgebildet. Ebenfalls dargestellt sind die Resultate der T_GWsim_ (Abb. 2). Die Beschreibung der Resultate bezieht sich auf den Beobachtungszeitraum Dezember 2019 bis Dezember 2020. Genannte Jahreszeiten folgen der meteorologischen Einteilung und sind definiert als Winter = Dezember–Februar (DJF), Frühling = März–Mai (MAM), Sommer = Juni–August (JJA) und Herbst = September–November (SON).

Die gemessenen T_TG_ in der Tiefgarage Storchen (Abb. 2 und Tab. 2) resultieren in einem Mittelwert von 19,6 °C, wobei die gemessenen Temperaturen mit einer Abweichung von 1,4 °C um das gleitende Tagesmittel der Messreihe variieren. Das absolute Maximum liegt bei 29,4 °C im August und das Minimum bei 14,2 °C im Dezember. Die durchschnittliche ∆T zu T_meteo_ (Mittelwert 12,2 °C) liegt bei 7,4 °C, wobei saisonale Unterschiede beobachtet werden können. Während die Abweichung im Winter durchschnittlich 10,4 °C beträgt, sind es im Frühling nur 5,2 °C, in den Sommermonaten nur noch 3,8 °C und im Herbst wieder 8,9 °C. T_GW_ an der GWM Bernoullianum (1052), welche sich 460 m nordwestlich und im Grundwasserzustrom der Tiefgarage befindet (Abb. 1 und Tab. 1), zeigt lediglich geringe Temperaturschwankungen zwischen 16,3 und 16,6 °C, mit einem Mittelwert von 16,4 °C. T_GW_-Maxima werden zwischen April und Juni erreicht, Minima im Dezember. Für T_GWsim_ resultierten ähnliche Werte, der Mittelwert beträgt 16,2 °C, allerdings zeigt die Temperatur hier deutliche saisonale Schwankungen mit einem lokalen Maximum um 16,3 °C im Winter und einem Minimum um 14,5 °C zu Beginn des Sommers bei anschließend starkem Anstieg bis auf 19,1 °C in den Sommermonaten.

**Table 2:** 

*Standort*	$\overline{T_{TG}}$^*a*^ *[°C]* *Jahr* Winter Frühling Sommer Herbst	$\overline{\Updelta T_{\text{TG float}}}$^b^ [°C]	$\overline{T_{\text{meteo}}}$^c^ [°C]	$\overline{\Updelta T_{\text{meteo}}}$^d^ [°C] *Jahr* Winter Frühling Sommer Herbst	$\overline{T_{\mathrm{GW}}\uparrow }$^e^ [°C] *Messstation*	$\overline{T_{\mathrm{GW}}\downarrow }$^f^ [°C]	$\overline{T_{\text{GWsim}}}$^g^ [°C]
*1: Storchen*	*19,6* 16,5 17,6 23,3 21,0	1,4	12,2	*7,4* 10,4 5,2 3,8 8,9	16,4 *1052*	–	16,2
*2: Clarastrasse*	*21,5* 20,2 20,8 22,8 22,4	0,2	12,2	*9,1* 14,2 8,4 2,7 10,3	14,1 *1064*	14,8 1161	15,8
*3: St. Claraspital, 2. UG*	*20,8* 17,6 20,0 25,0 22,0	1,3	12,2	*8,4* 11,5 7,6 5,0 9,9	14,1 *1064*	14,9 *3966* 14,8 *1075*	14,8
*3: St. Claraspital, 3. UG*	*19,6* 16,1 18,9 23,8 20,8	1,5		*7,1* 10,1 6,5 3,7 8,7			14,9
*4: St. Jakob-Park*	*19,6* 16,4 17,1 24,1 21,0	1,2	11,8	*7,0* 10,9 5,1 4,4 9,3	13,0 *20J96*	12,9 749	15,3
*5: Anfos*	*20,7* 17,9 18,8 23,2 22,8	0,3	11,8	*8,9* 12,3 6,8 3,4 11,1	16,7 *931*	18,9 *3755*	16,1

^a^Mittelwert T_TG_

^b^Durchschnittliche Abweichung T_TG_ zum gleitenden T_TG_-Tagesmittel

^c^Mittelwert T_meteo_

^d^Durchschnittliche Abweichung T_TG_ zu T_meteo_

^e^Mittelwert T_GW_ Grundwasserzustrom

^f^Mittelwert T_GW_ Grundwasserabstrom

^g^Mittelwert T_GWsim_

Mit 21,5 °C wird an der Clarastrasse der höchste T_TG_-Mittelwert gemessen, wobei hier auch die geringsten täglichen sowie gesamtzeitlichen Schwankungen zu beobachten sind (Abb. 2 und Tab. 2). Das absolute Maximum liegt bei 25,3 °C im August und das Minimum bei 19,4 °C im Dezember. Die durchschnittliche ∆T zu T_meteo_ (Mittelwert 12,2 °C) liegt bei 9,3 °C. Dabei betragen die durchschnittlichen Abweichungen vom gleitenden T_TG_-Mittelwert 0,2 °C. Die geringere Temperaturschwankung im Jahresverlauf bewirkt große bzw. kleine jahreszeitliche ∆T zwischen T_meteo_ und T_TG_ von 10,3 im Herbst, 14,2 im Winter über 8,4 im Frühling bis zu 2,7 °C in den Sommermonaten. Die beiden GWM befinden sich 1,6 km nordöstlich (Waldshuterstrasse 1064) und 140 m südwestlich (Dolderweg 1161) im Grundwasserzu- und -abstrom der Tiefgaragen (Abb. 1 und Tab. 1). Auch diese beiden Messstationen weisen keine großen Temperaturschwankungen des Grundwassers im Jahresverlauf auf. Bei GWM 1064 variiert das T_GW_-Tagesmittel zwischen 14,4 im Dezember und 13,6 °C im März mit einem Gesamtmittel von 14,1 °C. Sowohl im Winter als auch im Sommer werden höhere Temperaturen als im Frühling verzeichnet. Bei GWM 1161 schwankt das T_GW_-Tagesmittel zwischen 14,5 und 15,0 °C, ein klarer Trend über die Zeit ist hier nicht erkennbar. Der Mittelwert von 14,8 °C ist an dieser GWM um 0,7 °C höher als jener bei GWM 1064. T_GWsim_ beträgt an diesem Standort 15,8 °C und zeigt keine großen Abweichungen.

In der Tiefgarage St. Claraspital wurden auf zwei Stockwerken T_TG_-Messungen durchgeführt (2. UG und 3. UG; Abb. 2 und Tab. 2; aufgrund einer technischen Fehlfunktion wurden aus dem 3. UG nur Daten bis Mitte Oktober erfasst). Beide Temperaturkurven zeigen einen sehr gleichförmigen Verlauf, mit einer konstanten ∆T von ca. 1,2 °C. Die mittleren Temperaturen betragen hier 20,8 °C im 2. UG und 19,6 °C im 3. UG und sind damit um 8,6 bzw. 7,4 °C höher als der T_meteo_-Mittelwert (12,2 °C). Die Abweichungen vom gleitenden Tagesmittel fielen im höher gelegenen 2. UG etwas kleiner aus, durchschnittlich betrugen sie hier 1,3 °C, im 3. UG waren es rund 1,5 °C. Der Wertebereich der T_TG_-Tagesmittel liegt zwischen 30,4 und 13,5 °C im 2. UG bzw. zwischen 29,0 und 13,4 °C im 3. UG. Dies sind sowohl die höchsten als auch die tiefsten gemessenen Tagesmitteltemperaturen aller Standorte. Für den Standort St. Claraspital stehen die Datensätze von drei GWM zur Verfügung. Die GWM Waldshuterstrasse (1064) liegt 370 m nördlich im Grundwasserzustrom der Tiefgarage. Die GWM Magdenweglein 46 (3966) liegt 320 m südlich und die GWM Wettsteinallee 175 (1075) 500 m südwestlich, beide im Grundwasserabstrom der Tiefgarage (Abb. 1 und Tab. 1). Die T_GW_-Mittelwerte der Messstation südlich der Tiefgarage liegen mit 14,9 °C bei GWM 3966 und 14,8 °C bei GWM 1075 nah beieinander, wobei letztere in den Sommermonaten Minima von 14,5 °C erreicht, während die Temperaturabweichungen an GWM 3966 über den gesamten Verlauf der Aufzeichnung 0,1 °C nicht über- bzw. unterschreiten. Die T_GW_ bei GWM 1064 zeigt, wie bereits für den Standort der Tiefgarage Clarastrasse beschrieben, einen Temperaturverlauf mit Minima von 13,6 °C im Frühling. Mit einem Mittelwert von 14,1 °C liegt die Temperatur hier rund 1 °C unter den Mittelwerten der anderen beiden GWM. Für T_GWsim_ wurden die Simulationsergebnisse für die jeweiligen Messhöhen der beiden T_TG_-Messungen extrahiert, sie liegen bei 14,9 °C in der grundwassergesättigten und bei 14,8 °C in der grundwasserungesättigten Zone.

Die T_TG_-Messung des Standorts St. Jakob-Park zeigt einen ähnlichen Verlauf wie die Messungen in der Tiefgarage des St. Claraspitals (Abb. 2 und Tab. 2). Der Mittelwert beträgt 19,6 °C, 7,8 °C höher als der T_meteo_-Mittelwert (11,8 °C), das T_TG_-Tagesmittel bewegt sich zwischen 14,5 °C im Winter und 27,2 °C im Sommer. Die Abweichungen vom gleitenden Tagesmittel betragen durchschnittlich 1,2 °C. Die GWM G80 Karussell (20J96) befindet sich 1,1 km südsüdöstlich im Grundwasserzustrom der Tiefgarage St. Jakob-Park (Abb. 1). Der hier gemessene Mittelwert beträgt 13,0 °C, mit absoluten Minima von 12,6 °C im März und Maxima von 13,3 °C im September. Die mittlere ∆T zwischen T_TG_ und T_GW_ beträgt im Winter 3,4, im Frühling 4,1, im Sommer 11,1 und im Herbst 8,0 °C. Die zweite GWM (Redingstrasse 749) liegt 710 m nördlich im Grundwasserabstrom der Tiefgarage St. Jakob-Park (Abb. 1 und Tab. 1). Der T_GW_-Mittelwert beträgt hier 12,9 °C, 0,1 °C niedriger im Vergleich zu GWM 20J96. Die T_GW_ zeigen hier allerdings eine deutlich stärkere Schwankung über die Zeit, mit einem Maximum von 15,1 °C im Dezember und einem Minimum von 11,3 °C im Mai, liegen also zu Beginn der Messperiode um 2 °C höher als die T_GW_ bei GWM 20J96, unterschreiten diese allerdings im Februar, nähern sich ihnen ab Mai wieder an und überschreiten sie wiederum im August. Der Mittelwert der T_GWsim_ des Standorts St. Jakob-Park beträgt 15,3 °C, wobei der Verlauf von T_GWsim_ nur sehr schwache Temperaturschwankungen zeigt.

Der T_TG_-Mittelwert am Standort Anfos beträgt 20,7 °C, 8,9 °C höher als der T_meteo_-Mittelwert, wobei ∆T der Mittelwerte saisonalen Schwankungen ausgesetzt ist, von 12,3 im Winter über 6,8 °C im Frühling zu 3,4 °C im Sommer zu 11,1 °C im Herbst (Abb. 2 und Tab. 2). Die Temperaturschwankungen um das gleitende T_TG_-Tagesmittel betragen im Schnitt 1,1 °C. Auch für diesen Standort stehen die Daten von zwei GWM zur Verfügung: Die GWM Heuwaage (931) befindet sich ca. 430 m westlich im Grundwasserzustrom der Tiefgarage. Die mittlere T_GW_ beträgt hier 16,7 °C, mit einem Maximum von 19,2 °C im September und einem Minimum von 14,5 °C im März. ∆T zwischen T_GW_ und T_TG_ beträgt 4,0 °C. Die zweite GWM Brunngässlein (3755) liegt ca. 170 m nordöstlich im Grundwasserabstrom der Tiefgarage (Abb. 1 und Tab. 1). Der T_TG_-Mittelwert liegt hier bei 18,9 °C mit einem Maximum von 19,7 °C im November und einem Minimum von 18,2 °C im Mai. Die Temperatur ist hier durchschnittlich 2,2 °C höher als an der GWM 931, ∆T zwischen dem T_GW_ und T_TG_-Mittelwert beträgt 1,8 °C. Die T_GWsim_ resultiert am Standort Anfos in einem Mittelwert von 16,1 °C; auch hier ist, ähnlich wie für die Tiefgarage Storchen, ein Temperaturjahresgang zu erkennen, mit einem lokalen Maximum von 16,4 °C zum Ende des Winters und einem lokalen Minimum von 15,4 °C zum Sommerbeginn.

#### Korrelationen

Tab. 3 zeigt die Pearson-Korrelationskoeffizienten r der stündlichen Messungen von T_TG_ und T_meteo_. Die Korrelationsanalyse wurde für jeden Standort einmal vor und ein weiteres Mal nach dem Entfernen des linearen Trends, der durch den Temperaturanstieg über die Frühlings- und Sommermonate bedingt ist, durchgeführt. Die unveränderten Temperaturreihen weisen eine starke positive Korrelation mit Koeffizienten zwischen 0,86 und 0,75 auf. Nach Bereinigung des Trends resultiert der Korrelationskoeffizient in tieferen Werten zwischen 0,57 und 0,22, bleibt aber weiterhin im positiven Bereich. Für die weitere statistische Analyse wurden die durch den Trend unbeeinflussten Daten verwendet.

**Table 3:** 

Standort	Trend vorhanden	Trend entfernt	Zeitliche Auflösung	0	+24 h	+48 h	+72 h	+96 h	+120 h
1: Storchen	0,81	0,47	Stundenwerte Tagesmittel	0,44 0,48	*0,47* *0,52*	0,46 0,51	0,44 0,48	0,42 0,45	0,69 0,42
2: Clarastrasse	0,78	0,36	Stundenwerte Tagesmittel	0,29 0,37	0,34 0,42	*0,36* *0,45*	*0,36* *0,45*	0,35 0,44	0,34 0,43
3: St. Claraspital, 2. UG	0,86	0,57	Stundenwerte Tagesmittel	0,56 0,57	*0,57* *0,60*	0,54 0,55	0,51 0,52	0,49 0,49	0,44 0,43
3: St. Claraspital, 3. UG	0,85	0,53	Stundenwerte Tagesmittel	0,45 0,46	0,52 0,56	*0,53* *0,57*	0,52 0,56	0,52 0,55	0,47 0,49
4: St. Jakob-Park	0,75	0,32	Stundenwerte Tagesmittel	0,29 0,31	*0,32* *0,35*	0,31 0,34	0,31 0,33	0,30 0,32	0,29 0,31
5: Anfos	0,77	0,22	Stundenwerte Tagesmittel	0,15 0,15	0,19 0,20	0,20 0,22	0,21 0,23	*0,22* *0,23*	0,20 0,22

Tab. 3 zeigt auch die Resultate der Pearson-Korrelationsanalyse der trendbereinigten Temperaturreihen. Dabei wurden hier sowohl die Korrelationen der stündlichen Messwerte sowie die der diskreten Tagesmittelwerte verglichen. Zur Untersuchung, ob in den Tiefgaragen eine verzögerte Reaktion auf Temperaturschwankungen der atmosphärischen Temperaturen besteht, wurde die Analyse jedes Standortes wiederholt durchgeführt, bis eine maximale Korrelation erreicht wurde. Aus Tab. 3 geht hervor, dass eine solche Verzögerung existiert und, je nach Standort, zwischen einem und vier Tagen beträgt. Im Vergleich zwischen Stunden- und Tagesmittelwerten fallen die Korrelationskoeffizienten der Tagesmittel für jeden Standort höher aus als jene der stündlichen Messungen.

#### Temperaturmessung Nordtangente

**Figure 3:**
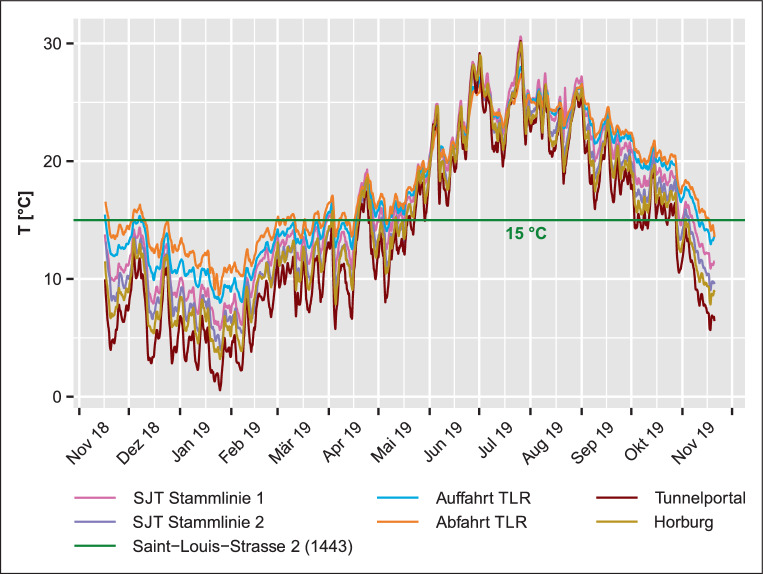

Die Mittelwerte der gemessenen Temperaturen im Tunnelbauwerk Nordtangente liegen zwischen 13,7 °C (Tunnelportal) und 18,1 °C (Abfahrt TLR), die tiefsten Temperaturmittelwerte wurden also in der Nähe der Einfahrt, die höchsten im Tunnelinneren gemessen. Die tiefsten Messwerte betragen −0,3 bis 7,7 °C, die Maxima liegen zwischen 28,4 und 32,8 °C. So entstehen sowohl in den Winter- als auch in den Sommermonaten Unterschiede zur Grundwassertemperatur von bis zu −15,3 bzw. 17,8 °C. Die Temperaturmessungen bilden im Jahresgang eine sinusförmige Kurve, die in etwa um die mittlere T_GW_ von ca. 15 °C oszilliert, mit einem Minimum im Winter, einem Maximum im Sommer und Schnittpunkten mit T_GW_ in den Frühlings- und Herbstmonaten (Abb. 3).

## Diskussion

### Korrelationen und Temperaturtagesgang

**Figure 4:**
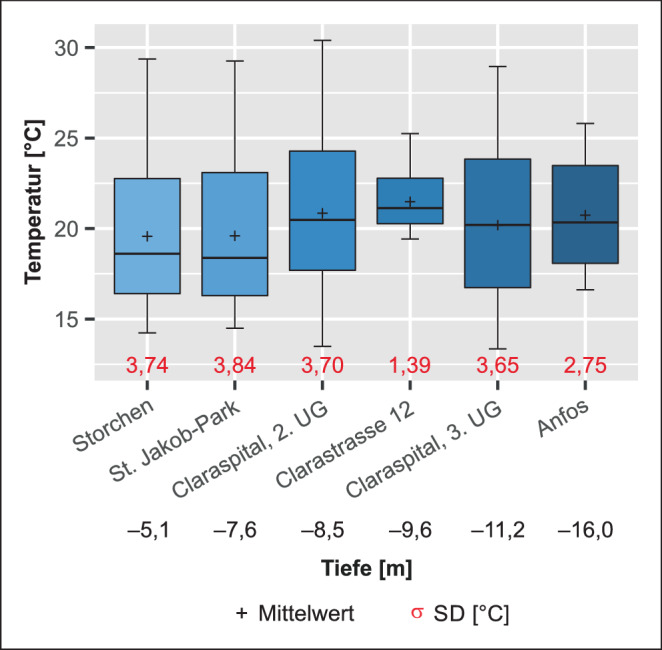

Die Resultate der ersten Korrelationsanalyse (Tab. 3) deuten darauf hin, dass die Schwankungen von T_TG_ sowohl dem Jahresverlauf als auch kurzfristigen Veränderungen von T_meteo_ folgen. Auf den ersten Blick spiegeln sich diese Schwankungen deutlich in den stündlichen Messungen der Tiefgaragen Storchen, St. Claraspital und St. Jakob-Park wider, mit täglicher ∆T von mehr als 2 °C vom Tagesmittelwert. Die tägliche ∆T der anderen beiden Standorte fallen geringer aus; in der Tiefgarage Anfos betragen diese 1,1 °C und am Standort Clarastrasse nur 0,1 °C. T_TG_ der Standorte St. Claraspital, Storchen und St. Jakob-Park weisen die größten Standardabweichungen auf (Tab. 4 und Abb. 4).

**Table 4:** 

Standort	Tiefe Messystem unter GOK [m]	$r_{h}$^a^ [–]	$r_{\overline{d}}$^b^ [–]	$tr_{\max }$^c^ [h]	$\overline{\Updelta T_{\text{GWfloat}}}$^d^ [°C]	σ^e^ [°C]
1: Storchen	−5,1	0,47	0,52	+24	2,1	3,74
2: Clarastrasse	−9,6	0,36	0,45	+48–72	0,1	1,39
3: St. Claraspital, 2. UG	−8,5	0,57	0,60	+24	3,7	3,70
3: St. Claraspital, 3. UG	−11,2	0,53	0,57	+48	3,2	3,65
4: St. Jakob-Park	−7,4	0,32	0,35	+24	2,6	3,84
5: Anfos	−16,0	0,22	0,23	+96	1,1	2,75

*GOK* Geländeoberkante

^a^Korrelationskoeffizient der stündlichen Messungen $r_{h}$

^b^Korrelationskoeffizient der Tagesmittel $r_{\overline{d}}$

^c^Verzögerung bis zum Eintreten des grössten Korrelationkoeffizienten $tr_{\max }$

^d^Durchschnittliche Abweichung von T_TG_ zum gleitenden Mittelwert $\Updelta T_{\text{GWfloat}}$

^e^Standardabweichung σ

**Figure 5:**
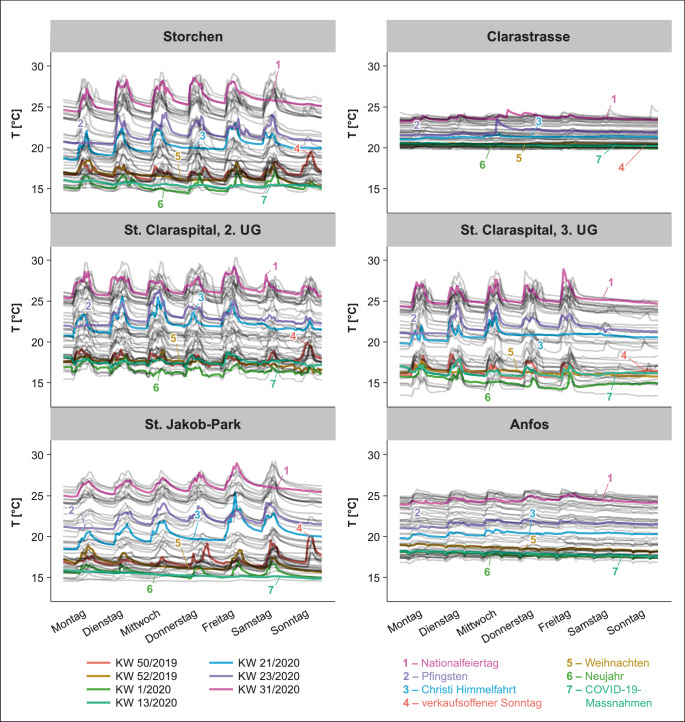

Dass die täglichen Temperaturschwankungen nicht von der Standorttiefe abhängig sind, zeigen u. a. die Werte der Messung im 3. UG der Tiefgarage des St. Claraspitals. Der mit 11,2 m Distanz zur Geländeoberkante zweittiefste Standort zeigt auch die zweithöchsten täglichen Temperaturschwankungen nach der Messung im 3. UG der Tiefgarage. Es muss also einen anderen Faktor als T_meteo_ geben, welcher die täglichen T_TG_-Ausschläge begründet und dazu führt, dass die Korrelationskoeffizienten der Stundenwerte tiefer ausfallen als jene der Tagesmittelwerte. Deshalb wurde eine genauere Analyse der Temperaturverläufe einzelner Wochen an den Standorten Storchen, St. Claraspital und St. Jakob-Park vorgenommen. Es wird deutlich, dass für diese Standorte tägliche T_TG_-Maxima an Wochenenden und Feiertagen fehlen bzw. schwächer ausgeprägt sind als jene an den Standorten Clarastrasse und Anfos. Dieses Phänomen zeigt sich besonders deutlich im Monat März. Aufgrund der COVID-19-Pandemie wurde am 16. März 2020 in der Schweiz die außerordentliche Lage gemäß Epidemiegesetz erklärt. Diese hatte zur Folge, dass, laut Medienmitteilung des Bundesamtes für Gesundheit (BAG) vom 13. März 2020, „alle Läden, Märkte, Restaurants, Bars sowie Freizeit- und Unterhaltungsbetriebe“ zwischen dem 16. März und dem 11. Mai 2020 geschlossen wurden (BAG 2020a). In diesem Zeitraum fallen die T_TG_-Schwankungen kleiner aus, im Vergleich zum T_meteo_-Tagesgang, der mehr oder weniger unverändert bleibt. Der größte beeinflussende Faktor ist dementsprechend die Nutzungsart der Tiefgaragen, also die Anzahl der ein- und ausfahrenden Fahrzeuge und der Betrieb von Kühl-, bzw. Heizanlagen, welche an Wochenenden und Feiertagen sowie während des „Lockdowns“ im Frühjahr deutlich geringer ausfallen. Am 18. Dezember 2020 erließ der Bundesrat eine erneute Verordnung zur Schließung von Restaurants, Freizeit‑, Sport und Kultureinrichtungen ab dem 22. Dezember 2020 (BAG 2020b). Einkaufsläden und einige Dienstleistungsbetriebe waren von den Maßnahmen nicht betroffen, demzufolge dieser zweite „Lockdown“ auch nur eine geringfügige Änderung der Tiefgaragennutzung bewirkte. Abb. 5 zeigt die täglichen Temperaturgänge in den Tiefgaragen, dargestellt in Kalenderwochen. In der Darstellung farblich hervorgehoben sind einige Wochen, die eine oder mehrere Anomalien wie z. B. Feiertage enthalten; konkret sind das einer der verkaufsoffenen Sonntage in der Adventszeit am 15.12.2019, die Weihnachtsfeiertage vom 25. bis 26.12.2019, Neujahr am 1. Januar 2020, eine Woche der durch den „Lockdown“ verordneten Betriebsschließungen vom 23. bis 29.03.2020, Christi Himmelfahrt am 21.05.2020, der Pfingstmontag am 01.06.2020 und der schweizerische Nationalfeiertag am 01.08.2020. Wie erwartet sind in den verschiedenen Tiefgaragen unterschiedliche Reaktionen zu beobachten.

Für die Tiefgarage Storchen spiegelt sich die vorwiegend von Kunden der umliegenden Läden und Geschäfte geprägte Nutzung im Verlauf von T_TG_ wider (Abb. 5). Obwohl die Tiefgarage an 365 Tagen im Jahr geöffnet ist, bleiben Temperaturschwankungen an allen gesetzlichen Feiertagen aus. Am verkaufsoffenen Sonntag ist hingegen eine deutlich höhere Temperatur zu verzeichnen als an den restlichen Tagen dieser Woche. Auch im Vergleich mit anderen Sonntagen ist der Unterschied signifikant. In der ausgewählten Woche während des „Lockdowns“ bleiben die sonst kennzeichnenden Temperatur-Peaks ebenfalls aus, sie ersetzt ein Tagesgang mit schwacher Fluktuation.

Die Temperaturschwankungen in der Tiefgarage Clarastrasse sind kaum vergleichbar mit denjenigen der Tiefgarage Storchen (Abb. 5). Unterbrochen von einigen Ausreißern zeigt sich, dass Temperaturschwankungen in wärmeren Wochen stärker ausfallen als in kälteren. Aufgrund der mehrheitlich privaten Nutzung der Tiefgarage bleibt der verkaufsoffene Sonntag ohne Auswirkung auf T_TG_. Weihnachten und Neujahr sind nicht von anderen Tagen zu unterscheiden und auch die ausgewählte Woche während des „Lockdowns“ unterscheidet sich kaum von anderen Wochen.

Die beiden Messungen in der Tiefgarage des St. Claraspitals zeigen einige Unterschiede (Abb. 5). Während sich im 2. UG, welches überwiegend von Besuchenden der Patienten genutzt wird, auch an Sonn- und einigen Feiertagen, wie Christi Himmelfahrt und dem 1. August, Temperatur-Peaks entwickeln, bleibt die Temperatur im Stockwerk darunter, welches v. a. dem Personal zur Verfügung steht, meist über das gesamte Wochenende, ebenso wie an Feiertagen, vergleichsweise konstant. Da das St. Claraspital als Gesundheitseinrichtung nicht dem „Lockdown“ unterlag, sind in dieser Zeit im 3. UG keine ungewöhnlichen Abweichungen der Temperatur zu beobachten, im 2. UG fallen die täglichen Schwankungen aufgrund der strengeren Regeln für Besuchende etwas tiefer aus.

Die Tiefgarage St. Jakob-Park ist wie die Tiefgarage Storchen von umliegenden Geschäften und einem angrenzenden Shoppingcenter geprägt. Ein Tagesgang, welcher von Montag bis Samstag deutlich zu erkennen ist, fällt an Sonn- und gesetzlichen Feiertagen komplett weg. Der verkaufsoffene Sonntag zeigt eine deutliche Temperaturerhöhung. In der Zeit des „Lockdowns“, in welcher bis auf einen Lebensmittelladen alle Geschäfte des Shoppingcenters schließen mussten, verschwinden die Temperaturschwankungen fast gänzlich.

Der Temperaturverlauf in der Tiefgarage Anfos lässt sich am ehesten mit jenem in der Tiefgarage Clarastrasse vergleichen, obwohl der Unterschied zwischen Wochen- und Wochenendtagen hier noch stärker ausfällt, v. a. in Wochen mit höheren Temperaturen. Die Tiefgarage ist zwar generell durch die Öffentlichkeit nutzbar, das Stockwerk, in welchem die Messungen erfasst wurden, ist jedoch durch ein geringes Verkehrsaufkommen gekennzeichnet. Fehlende Temperaturmaxima an Pfingsten und Christi Himmelfahrt sind sehr leicht ausgeprägt, in Wochen, welche durch kalte Temperaturen geprägt sind, sind keine täglichen Ausschläge nach oben mehr zu erkennen. Stattdessen wird ein regelmäßiges Muster im Verlauf von T_TG_ erkennbar, nach welchem die Temperatur zweimal pro Tag um ca. 0,1 bis 0,2 °C absinkt. Nach Angaben des technischen Dienstes der Tiefgarage handelt es sich dabei um die Einflüsse einer Lüftungsanlage, welche in einem zwölfstündigen Rhythmus kurzzeitig eingeschaltet wird, um Frischluft in die Tiefgarage zu leiten. Die kalten Außentemperaturen im Winter sorgen für eine kurzzeitige Abkühlung in der Tiefgarage.

Zusammenfassend lassen sich folgende Erkenntnisse ableiten: (1) je höher die Ausgangstemperatur desto höher ist auch die Ausprägung täglicher Temperaturschwankungen; (2) das Ausbleiben von Temperaturausschlägen an Sonn- und Feiertagen in den Tiefgaragen, welche ansonsten die ausgeprägtesten Tagesgänge zeigen, deuten darauf hin, dass hier die Nutzung, also das Einstellen aufgeheizter Fahrzeuge und der Betrieb von Heiz- und Kühlanlagen, den stärksten Einfluss auf die Temperaturen hat.

Wie bereits beschrieben erklärt sich dadurch auch die Ungleichheit der Korrelationskoeffizienten von Stundenwerten und Tagesmitteln. Die nutzungsbedingte tägliche Temperaturfluktuation steht nicht in direktem Zusammenhang mit dem Tagesgang von T_meteo_ und resultiert somit in einer geringeren Korrelation der stündlichen Messungen. Eine erneute Bereinigung der Temperaturreihen durch das Entfernen der täglichen, „anthropogen verursachten“ Temperatur-Peaks könnte die Korrelation für die Tagesmittelwerte weiter verbessern.

Werden die T_TG_-Tagesmittelwerte betrachtet, gewinnt der Faktor Standorttiefe wieder an Bedeutung (Tab. 4). Mit zunehmender Tiefe der Messstation nimmt auch die Verzögerung bis zum Erreichen des größten r‑Werts zu. Auch hier kann der Einflussfaktor der Tiefgaragennutzung nicht vernachlässigt werden, was sich erneut am Beispiel der Tiefgarage des St. Claraspitals zeigen lässt: Im 3. UG (−11,2 m) wird der höchste r‑Wert nach 48 h festgestellt, in der höher gelegenen Tiefgarage Clarastraße (−9,6 m) zwischen 48 und 72 h, im wiederum höheren 2. UG der Tiefgarage des St. Claraspitals (−8,5 m) nach 24 h. Dementsprechend ist eine Kombination der Faktoren Tiefe und Nutzungsintensität für die Reaktionsdauer des T_TG_-Tagesmittelverlaufs verantwortlich. Die deutlich größeren Verzögerungen von bis zu 96 h treten in den wenig genutzten Tiefgaragen auf, was darauf hindeutet, dass auch die Reaktionszeiten hier, entsprechend ihrer Tiefe, ohne die nutzungsbedingten Temperaturschwankungen länger ausfallen würden.

Zusammenfassend zeigen die Korrelationsanalysen den Zusammenhang der meteorischen Lufttemperatur mit jener in den Tiefgaragen. Dabei zeigt der Temperaturverlauf in den Tiefgaragen eine klare Abhängigkeit von der Nutzungsart: In Tiefgaragen mit höherem Aufkommen täglicher Ein- und Ausfahrten konnten größere tägliche Temperaturanstiege nachgewiesen werden, durch welche Unterschiede von bis zu 2 °C in den Tagesmittelwerten zu verzeichnen sind. Besonders deutlich konnte dies im Zeitraum des „Lockdowns“ während der COVID-19-Pandemie zwischen März und Mai 2020 beobachtet werden. So blieben für Tiefgaragen, welche durch die Öffentlichkeit genutzt werden (z. B. das Parking eines Shoppingcenters), regelmäßig eintretende tägliche Temperaturschwankungen aus. Wenig Veränderungen konnten für Tiefgaragen, welche privat genutzt oder Teil einer essenziellen Einrichtung (z. B. angeschlossen an ein Spital) waren, verzeichnet werden.

### Einfluss von T_TG_ auf T_GW_

**Figure 6:**
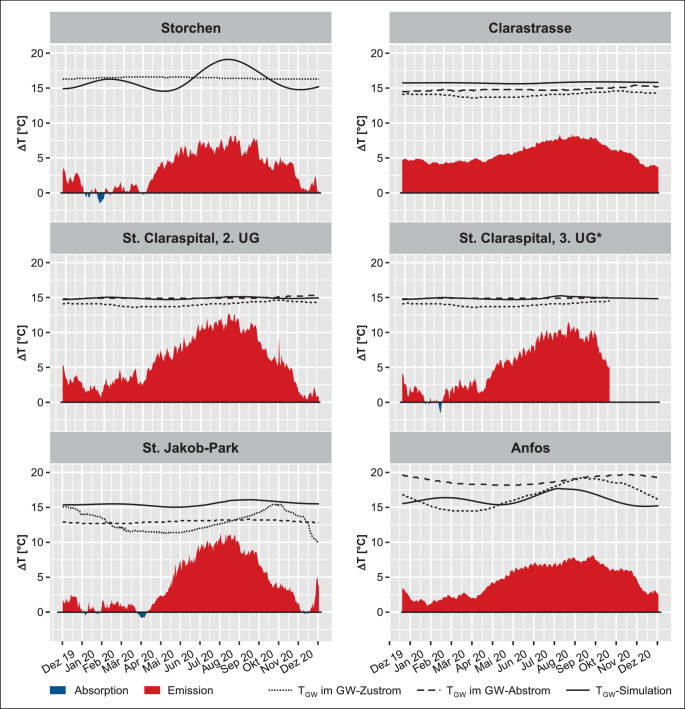

In den Temperaturzeitreihen ist deutlich zu erkennen, dass T_TG_ mehrheitlich T_GW_ überschreiten. Zur genaueren Analyse der Temperaturunterschiede wurde ∆T der Tagesmittel von T_TG_ und T_GW_ sowie den T_GWsim_ als Flächendiagramme dargestellt (Abb. 6). Positive Werte deuten darauf hin, dass T_TG_ die T_GW_ überschreitet und dementsprechend die Tiefgarage Wärme nach außen emittiert; bei negativen Werten unterschreitet T_TG_ die T_GW_, die Tiefgarage absorbiert Wärme. Um die Schwankungen von T_GW_ und T_GWsim_ in den Vergleich miteinzubeziehen werden diese Temperaturkurven ebenfalls als Referenz dargestellt.

Abb. 6 zeigt für den Standort Storchen die oft großen saisonalen Unterschiede in den Winter- und Frühlingsmonaten. ∆T, abgeleitet aus T_TG_ in Relation zu T_GW_ (GWM 1052) und T_GWsim_, schwankt zwischen 3 und −1 °C. Wärmeemission und -absorption gleichen sich bis Ende April mit einer durchschnittlichen Temperatur von ca. 16,2 °C in etwa aus. In den Sommermonaten wird ∆T deutlich größer, fällt nicht mehr in den negativen Bereich und erreicht ein Maximum von 8,2 °C über T_TG_. T_GWsim_ bildet über die Zeit eine sinusförmige Kurve mit einer Amplitude von 0,9 °C, mit einem lokalen Maximum im Mai, einem lokalen Minimum im Februar und einem weiteren Maximum bei 19,1 °C im September.

Im Vergleich zum Standort Storchen zeigen die Temperaturschwankungen der Tiefgarage Clarastrasse eine deutlich geringere Fluktuation. ∆T variiert hier zwischen 3,6 °C im Winter und 8,4 °C im Sommer, bleibt also stets im positiven Bereich. Der T_GWsim_-Mittelwert beträgt 15,8 °C. Ähnlich sieht es für die beiden GWM aus: An GWM Waldshuterstrasse (1064) wurde mit durchschnittlich 14,0 °C eine tiefe T_GW_ gemessen, ∆T liegt zwischen 5,7 und 10,2 °C; die T_GW_ an GWM Dolderweg (1161) liegt dazwischen, mit einem Mittelwert von 14,7 °C und einer Variation zwischen 5,0 und 9,5 °C.

Die in unterschiedlichen Tiefen gemessenen T_TG_ in der Tiefgarage des St. Claraspitals unterscheiden sich untereinander, wie in den Temperaturzeitreihen bereits zu erkennen war (Abb. 2). Ein ∆T von 1,3 °C zwischen dem 2. und 3. UG bewirkt, dass das mit T_GWsim_ abgeleitete ∆T im unteren Stockwerk im Januar kurzzeitig negative Werte annimmt, mit einem Minimum von −1,5 °C. Eine in etwa ausgeglichene Bilanz zwischen Wärmeemission und -absorption besteht aber nur im Januar; unmittelbar vorher und direkt anschließend nehmen die Temperaturen wieder zu und ∆T erreicht Maximalwerte von 11,3 °C. Gleiche Werte erreicht ∆T für die GWM Magdenweglein 46 (GWM 3966), an der GWM Waldshuterstrasse (1064) sind diese mit 12,5 °C noch höher. Die höhere Temperatur im 2. UG führt dazu, dass ∆T hier gänzlich im positiven Bereich liegt, also permanent Wärme von der Tiefgarage nach außen emittiert wird. Die größten ∆T-Werte liegen hier bei 12,8 (GWM 3966) bzw. 13,5 °C (GWM 1064). Der Messpunkt im 2. UG liegt oberhalb des Grundwasserpegels, hier wird die Wärme an das umliegende Lockergestein und nicht an das Grundwasser abgegeben. Im Umkehrschluss bedeutet dies, dass auch kein Grundwasser auf Höhe des 2. UG fließt, welches die Wärme aufnehmen und wegtransportieren kann (advektiver Wärmetransport); ein weiterer Faktor, neben der verstärkten Nutzung, der den Temperaturunterschied zwischen den Untergeschossen erklärt.

Für die Tiefgarage St. Jakob-Park nimmt das mit T_GWsim_ abgeleitete ∆T in den Winter- und Frühlingsmonaten vergleichsweise kleine Werte an. Anfang April wurden bei einem lokalen Temperaturminimum sogar negative Werte verzeichnet; mit dem Ansteigen der Temperaturen in den Sommermonaten nimmt auch ∆T wieder zu und erreicht Werte bis 11,3 °C. Der Mittelwert beträgt 15,3 °C. Die T_GW_-Mittelwerte betragen an der GWM G80-Karussell (20J96) 13,0, an GWM Redingstrasse (749) 12,8 °C, allerdings unterscheiden sich die ∆T-Werte aufgrund der verschiedenen Verlaufskurven; T_GW_ in GWM 749 sinkt dabei über die Zeit um 3,6 °C, weshalb die ∆T-Werte im Winter deutlich tiefer ausfallen als im Sommer. T_GW_ in GWM 749 übersteigt T_TG_ in den Sommermonaten um bis zu 14,1 °C, die T_GW_ in GWM 20J96 um maximal 13,9 °C.

Die Darstellungen in Abb. 6 zeigen, dass T_TG_ während überwiegender Zeit höher sind als jene des umgebenden Grundwassers. Vor allem in den Sommermonaten sind sehr hohe Temperaturunterschiede von bis über 14 °C zu beobachten. Auch im Winter und Frühling sinkt ∆T selten in den negativen Bereich und dies auch nur, wenn bereits deutlich erhöhte T_GW_ vorliegen. Die Wärmeabsorption kann also die -emissionen der Tiefgaragen nicht ausgleichen; über den Zeitraum, für den T_GW_-Messungen vorliegen, beläuft sich die höchste Anzahl von Tagen mit negativem ∆T-Wert auf 32 (Tab. 5).

**Table 5:** 

Standort	*n* $\Updelta \mathrm{T}< 0$ [#]	*n* $\Updelta \mathrm{T}> 0$ [#]	$n_{\text{total}}$ [#]	Verhältnis $n_{\text{total}}$ [#]
1: Storchen	32	334	366	~ 0,09
2: Clarastrasse	0	366	366	–
3: St. Claraspital, 2. UG	0	366	366	–
3: St. Claraspital, 3. UG	13	311	324	~ 0,04
4: St. Jakob-Park	28	338	366	~ 0,07
5: Anfos	0	366	366	–
Nordtangente	191	174	365	~ 0,52

Zusammenfassend zeigen die Vergleiche zwischen den Temperaturunterschieden in den Tiefgaragen, den Temperaturmessungen in nahegelegenen Grundwassermessstellen und den Resultaten einer Wämetransportmodellierung den Zusammenhang von Einwirkungen der Nutzungsintensität der Tiefgaragen. In Abhängigkeit von Gebäudetiefe, Grundwasserspiegelhöhe und -fließgeschwindigkeiten konnten die Wärmelasten für die ausgewählten Tiefgaragen quantitativ abgeschätzt werden. Dabei zeigte sich, dass bereits signifikante Unterschiede von mehr als 1 °C entstehen, je nachdem, ob ein Stockwerk im grundwassergesättigten Bereich des Untergrunds liegt oder in der grundwasserungesättigten Zone. V. a. auch die Flächengröße, welche in Kontakt mit dem Grundwasser steht, spielt eine große Rolle, wie viel Wärme in das Grundwasser abgegeben wird.

### Vergleich Tiefgaragen – Autobahntunnel Nordtangente

Im Gegensatz zu den Tiefgaragen liegen die Temperaturen in den Tunneln der Nordtangente an durchschnittlich 191 Tagen der einjährigen Messperiode unter der mittleren T_GW_, also während ca. 52 % der Zeit. In den Winter- und teilweise auch in den Herbst- und Frühlingsmonaten wurden Temperaturen gemessen, die die mittlere T_GW_ deutlich um bis zu 15 °C unterschreiten. In diesen Monaten wird demnach Wärme aus dem Grundwasser durch die Untergrundstruktur absorbiert. In den Sommermonaten kehrt sich die Situation, die Temperaturen steigen um bis zu 17,8 °C über die mittleren T_GW_, während eines Zeitraums von durchschnittlich 174 Tagen wird Wärme von den Tunnelbauwerken an den Grundwasserkörper abgegeben (Tab. 5).

Es entsteht also über das gesamte Jahr hinweg ein Gleichgewicht zwischen Wärmeemission und -absorption, welches in den Tiefgaragen nicht zu erkennen ist. Die Gebäudeform der Untergrundstruktur spielt also eine wesentliche Rolle im Wärmehaushalt: Die offene Struktur des Tunnelbauwerkes lässt vor allem über die Tunnelportale einen deutlich größeren Austausch mit der Außenlufttemperatur und demnach ausgeprägtere Temperaturschwankungen über die Jahreszeiten zu als die eher geschlossene, kompaktere Form der Tiefgarage. Im Tunnel besteht zudem eine ständige Bewegung der Fahrzeuge sowie eine zusätzliche künstliche Ventilation, welche den Luftaustausch fördert. Dagegen werden die Fahrzeuge in den Tiefgaragen meist abgestellt, wo sie abkühlen und Wärme an die Umgebung abgeben.

**Figure 7:**
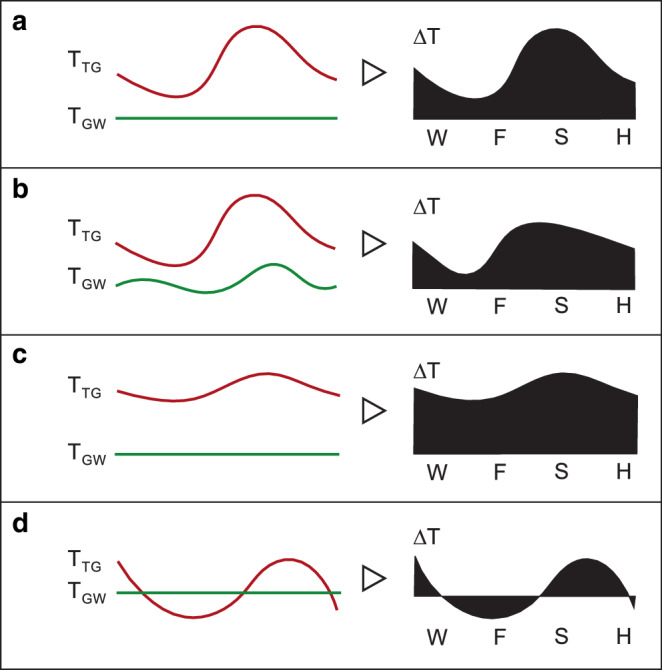

### Konzept ∆T-Profile für den thermischen Einfluss von Untergrundstrukturen

Für die untersuchten Untergrundstrukturen, einschließlich der Tiefgaragen und des Autobahntunnels Nordtangente, können vier verschiedene thermische Einwirkungsmuster auf das Grundwasser abgeleitet werden (Abb. 7):

1. Tiefgaragen St. Claraspital und St. Jakob-Park: T_TG_ mit hoher Fluktuation, tief im Winter und hoch im Sommer; T_GW_ verläuft tief und flach. ∆T im Winter mit kleinen/negativen Werten, die im Verlauf des Jahres steil zu- und wieder abnehmen.
2. Tiefgaragen Storchen und Anfos: T_TG_ mit hoher Fluktuation, tief im Winter und hoch im Sommer; T_GW_ verläuft hoch, mit versetzter Temperaturkurve zu T_TG_. ∆T zu Beginn im Winter mit kleinen/negativen Werten, die im Jahresverlauf steil zu- und stetig wieder abnehmen.
3. Tiefgarage Clarastrasse: T_TG_ mit geringer Fluktuation, hoch im Winter und noch höher im Sommer; T_GW_ verläuft hoch und flach. ∆T durchgehend mit mittleren, im Verlauf leicht fluktuierenden Werten.
4. Tunnelbauwerk Nordtangente: Temperatur der Tunnelinnenluft mit sehr hoher, saisonaler Fluktuation; T_GW_ verläuft hoch und flach (Abb. 3). ∆T nimmt im Gegensatz zu den Tiefgaragen über größere Zeiträume negative Werte an.

### Wärmelast der Untergrundstrukturen

Die Größenordnung der Wärmelast, die von den unterirdischen Gebäudestrukturen sowohl in die ungesättigte als auch die gesättigte Zone entweicht, kann mit einer einfachen Berechnung quantitativ abgeschätzt werden (Epting et al. 2013). Unter Berücksichtigung der Gebäudefläche $A_{GW}$, welche mit dem Grundwasser der umliegenden gesättigten bzw. dem Gestein/Boden der ungesättigten Zone in Kontakt steht, des Wärmedurchgangskoeffizienten $k$ der Gebäudeaußenwände und des Temperaturunterschieds ∆T zwischen der Temperatur in der Untergrundstruktur und den Grundwassertemperaturen im Zustrom (in K) kann der Wärmeaustausch$E$ berechnet werden:

2 $$E=k\cdot A_{GW}\cdot \Updelta T$$

Das älteste der untersuchten Gebäude ist die Tiefgarage Storchen (Baujahr 1959), weshalb basierend auf dem Normenwerk des Schweizerischen Ingenieur- und Architektenvereins (SIA 380/1) für alle Tiefgaragen eine gute Gebäudeisolation und demnach ein Wärmedurchgangskoeffizient k von rund 0,3 W m^−2^ K^−1^ angenommen werden kann. Ausgehend von den Daten zu Gebäudegrundflächen und den gemessenen Stockwerkshöhen lassen sich diese Berechnungen auf die Standorte dieser Arbeit übertragen. Dazu wurden die jeweiligen Flächen der Seitenwände der Tiefgaragen, die unterhalb des Grundwasserspiegels liegen, berechnet. In den Fällen, in welchen die Gebäudeunterkante ebenfalls im Grundwasser liegt und nicht in den anstehenden Fels gebaut wurde, werden die Grundfläche addiert und die Gesamtflächen mit den Wärmelasten pro m^2^ verrechnet. Für jede Tiefgarage konnten somit die Wärmelasten des ∆T-Gesamtmittelwerts sowie vergleichsweise kleinere ∆T-Werte der Winter- und größere ∆T-Werte der Sommermonate berechnet werden (Tab. 6), um saisonale Unterschiede aufzeigen zu können.

**Table 6:** 

*Standort*	*A*_*GW*_* [m*^*2*^*]*	*E*_*norm*_* [W m*^*−2*^*]*			*E*_*ges*_* [kW]*		
		Mittel	Winter	Sommer	Mittel	Winter	Sommer
1: Storchen	29.995	0,93	0,0	2,41	28,0	0,0	72,4
2: Clarastrasse	4041	0,30	0,14	0,36	9,0	7,5	11,0
3: St. Claraspital	4905	0,30	0,10	0,50	9,1	3,9	15,1
4: St. Jakob-Park	59.802	3,96	2,18	6,95	118,9	65,5	208,4
5: Anfos	12.155	0,54	0,28	0,75	16,1	8,5	22,5
Nordtangente	111.000	1,2	−6,54	8,78	36,9	−196,4	263,5

**Figure 8:**
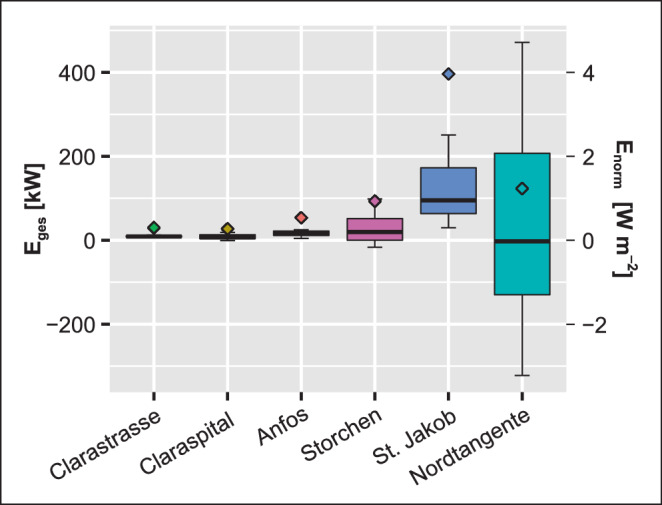

Tab. 6 und Abb. 8 zeigen, dass das Ausmaß des Wärmeaustausches nicht zwangsläufig mit der Größe des Gebäudes zusammenhängt, sondern mit der Kontaktfläche zur grundwassergesättigten Zone. Die Resultate der potenziellen Wärmeflüsse der Tiefgaragen sind modellhaft zu betrachten, da zusätzliche beeinflussende Faktoren auf die Temperaturen wie die Auswirkung des Grundwasserregimes nicht berücksichtigt wurden.

Es ist schwierig, die Wärmelasten verschiedener Städte mit unterschiedlichen städtischen Strukturen miteinander zu vergleichen. Die in Epting et al. (2017b) abgeleiteten Wärmelasten von Untergrundstrukturen in Basel liegen zwischen 0,2 und 0,9 W m^−2^, abhängig von der Lage der Strukturen innerhalb des Aquifers sowie von den hydraulischen und baulichen Randbedinungen. In Menberg et al. (2013a) resultierten aus einer räumlichen Analyse der Wärmelasten von Untergrundstrukturen Werte im Bereich von −0,1 und > 10 W m^−2^. In Benz et al. (2015) werden anthropogene Wärmelasten von Untergrundstrukturen in der Größenordnung von 3,61 ± 3,37 W m^−2^ für Karlsruhe und von 0,57 ± 0,47 W m^−2^ für Köln vorgestellt. Rees et al. (2000) und Thomas und Rees (1999) dokumentieren Wärmelasten durch Erdgeschossplatten von Gebäuden zwischen 0 und 20 Wm^−2^ und Ferguson und Woodbury (2004) schätzten den Wärmeverlust unter einem Gebäude auf ~ 2 W m^−2^. Die im Rahmen dieser Untersuchungen abgeleiteten Wärmeeinträge liegen also durchaus im Erfahrungsbereich vorangegangener Untersuchungen.

An Orten mit großer Grundwassermächtigkeit und hohen Grundwasserfließgeschwindigkeiten ist zu erwarten, dass mehr Wärme von den Tiefgaragen abgeführt wird (advektiver Wärmetransport). Am Beispiel der Tiefgaragen Storchen und Anfos in der Großbasler Innenstadt sowie St. Claraspital am Kleinbasler Stadtrand wird dies deutlich. Die Grundwasserfließgeschwindigkeiten werden hier durch Spundwände am Rheinufer, welche die Fluss-Grundwasser-Interaktion verhindern sollen, stark abgemindert (Epting et al. 2017b). Durch die verringerte Strömung im Grundwasserkörper werden die Temperatureinträge nur langsam abgeführt, T_GWsim_ unterscheidet sich an der Tiefgarage Storchen mit 16,2 °C, bzw. mit 16,1 °C an der Tiefgarage Anfos nur geringfügig von T_GW_ der GWM 1052 mit 16,5 °C im Grundwasserzustrom der Tiefgarage Storchen bzw. ist sogar tiefer als T_GW_ mit 18,6 °C an der GWM 3755, die im Grundwasserabstrom der Tiefgarage Anfos liegt; vor dem Rheinufer entsteht somit ein Wärmestau (Abb. 1). Im Gegensatz dazu folgt der Grundwasserfluss um die Tiefgarage St. Claraspital einem hydraulischen Gefälle von ca. 0,5 %. T_GWsim_ liegt hier bei 14,9 °C und um knapp ein Grad höher als T_GW_ im Grundwasserzustrom; im Grundwasserabstrom bei GWM 1075, die in ihrer Position leicht lateral verschoben zum Fließrichtungsvektor des Grundwassers liegt, erreicht die Temperatur nur 14,8 °C, ist also wieder geringfügig gesunken.

## Schlussfolgerungen

Eine nachhaltige thermische Bewirtschaftung urbaner Untergrundressourcen sollte eine angemessene Bewertung der thermischen Auswirkungen, einschließlich der Quantifizierung der Wärmelasten von Gebäudestrukturen, umfassen. Im Rahmen unserer Untersuchungen können wir die folgenden Schlussfolgerungen für die evaluierten Untergrundstrukturen in Basel ziehen:

- Zu fast jedem Zeitpunkt liegt T_TG_ über T_meteo_, erst in den Frühlings- und Sommermonaten liegt T_meteo_ aufgrund der hohen Außenlufttemperaturen kurzzeitig über T_TG_. In allen Temperaturzeitreihen, welche in den Tiefgaragen aufgezeichnet wurden, ist mit dem Zeitverlauf von Winter zu Sommer ein Anstieg der Temperaturen zu beobachten, je nach Standort mit unterschiedlicher Ausprägung.
- Die abgeschlossene Gebäudeform von Tiefgaragen begünstigt das Entstehen erhöhter T_TG_, welche in einer mehr oder weniger konstanten Wärmelast in das Grundwasser resultieren. Darin unterscheiden sich Tiefgaragen von Tunnelbauwerken, die deutlich größere jahreszeitliche Temperaturunterschiede und einen vergleichsweise ausgeglicheneren Wärmeaustausch aufweisen.
- Die Resultate illustrieren den thermischen Einfluss der Untergrundstrukturen auf das Grundwasser, wobei die Temperaturen im Grundwasserabstrom der Tiefgaragen teilweise um bis zu 2,7 °C höher sind verglichen mit jenen in den GWM gemessenen T_GW_ im Grundwasserzustrom.
- Ein wesentlicher Einflussfaktor auf T_TG_ ist der Wärmeeintrag von Kraftfahrzeugen, der Einflussfaktor Nutzung ist größer als jener der Standorttiefe. Dieser sorgt für eine zusätzliche Erhöhung der T_TG_-Tagesmittel um 1 bis 2 °C.

## Abkürzungen

$A_{GW}$: Gebäudefläche im Kontakt mit dem Grundwasser
BAES: Basel Aeschenplatz
BKLI: Basel Klingelbergstrasse
$$E$$: Wärmeaustausch
GW: Grundwasser
GWM: Grundwassermessstelle
$$k$$: Wärmedurchgangskoeffizient
SUHI: Subsurface Urban Heat Island
T_GW_: Grundwassertemperatur
T_GWsim_: simulierte Grundwassertemperatur
T_TG_: Temperatur der Tiefgaragenmessungen
T_meteo_: meteorische Temperatur
∆T: Temperaturdifferenz
